# Information flow in the pharmaceutical supply chain

**Published:** 2015

**Authors:** Nazila Yousefi, Ahmad Alibabaei

**Affiliations:** a *Department of Pharmacoeconomics and Pharma Management, School of Pharmacy, Shahid Beheshti University of Medical Sciences, Tehran, Iran. *; b *Department of Industrial Safety, School of Health, Safety and Environment, Shahid Beheshti University of Medical Sciences, Tehran, Iran. *

**Keywords:** information systems, supply chain management, Iran, pharmaceutical supply chain

## Abstract

Managing the supply chain plays an important role in creating competitive advantages for companies. Adequate information flow in supply chain is one of the most important issues in SCM. Therefore, using certain Information Systems can have a significant role in managing and integrating data and information within the supply chain. Pharmaceutical supply chain is more complex than many other supply chains, in the sense that it can affect social and political perspectives. On the other hand, managing the pharmaceutical supply chain is difficult because of its complexity and also government regulations in this field. Although, Iran has progressed a lot in pharmaceutical manufacturing, still there are many unsolved issues in managing the information flow in the pharmaceutical supply chain. In this study, we reviewed the benefits of using different levels of an integrated information system in the supply chain and the possible challenges ahead**.**

## Introduction

The term Supply chain is increasingly being applied instead of logistics and includes planning, sourcing, production and distribution (1). Supply chain evolution includes three periods; the first period is “inventory push” (1960-1975), in which the main focus is storage and financial management. The Second period is “customer pull” (1975-1990) focusing on customer attraction and product management and the third period is supply chain management, focusing on supply chain and managing relationships with customers. Supply chain management (SCM) which is integrating the key business processes from original suppliers to end users in a way that can create value for customers and stakeholders, initiated first from the Toyota Company, by switching their method in inventory management from “inventory push” to the “Just In Time” method (2). SCM can provide inter and intra integration by streaming information in and out of an organization (3). previously, supply chain definition consisted of three steps; procurement, production and distribution(4) which is then changed to three main flows: material, funds and information (5).Since without information flow there is not any product flow, Information flow is considered the most important one (1).Information sharing and information management which is necessary for coordination and collaboration among supply chain partners can reduce the supply chain risks (6). For example, the most famous issues in supply chain are called “Whip bull effect” and “illusive stock”;that lead to customer dissatisfaction, high stock in warehouses and high rate of returned products and transportation costs, and happen due to the lack of the right information at the right time (7).Information systems by reducing "silo effect" can prevent these problems by providing the right information in the right time for the right decision maker(8). In addition, information systems are integral parts of agile supply chain which can increase the speed and flexibility of it (9).

A well managed supply chain is considered as an important competitive advantage, but in recent years, by increase in the importance of information, a supply chain information system (SCIS) can be considered as the main competitive advantage.

SCIS can be useful in synchronizing all the activities of the entirety of active members of a supply chain including production, storage and shipment, and can also help in forecasting future demands and proper planning (2). SCIS is able to increase operational efficiency, effectiveness and flexibility, improve customer services, and reduce both costs and problems (10). These benefits of SCIS are followed by higher accuracy, faster processing, higher visibility and immediate availability (1).

Pharmaceutical supply chain is very complex, since pharmaceuticals are vital products and availability and accessibility of them are important issues for both companies and governments. It is crucial that drugs be delivered at the right time to the right person in standard conditions. Improper distribution of drugs, not only affects companies' reputation, costumers' satisfaction and companies’ profit, but also could also distributes the healing processes of patients and produces negative effects on public health.

In addition to different natures of the goods which are being delivered through pharmaceutical supply chains, there are some other specific issues in pharmaceutical supply chains including tight regulations, reimbursement, pricing applied by government agencies, direct sell models, 3PLs (third party logistics), product diversity, hardness in forecasting a product’s life cycle, shipment of R&D products for clinical studies and counterfeit products (11) which all lead to putting the pharmaceutical supply chain in a different category called “life science” and “healthcare supply chain (LSHC)”.

The importance of managing the pharmaceutical supply chain and its unique characteristics, made us to conduct this review.

## Experimental

*Methods*

This review article was performed in 2012 in order to explain the basics of information systems in pharmaceutical supply chains, and also to elucidate the applications of transaction systems, management control systems, “decision analysis” and strategic planning systems. We will complement the paper with a discussion on the SCIS which is being developed in Iran’s pharmaceutical supply chain.

*Findings*

Information systems in supply chains can be divided into four layers. Although, applying all the levels is not necessary, using all of them can be recommended in pharmaceutical supply chain.

Transaction level is the heart of an SCIS, which initiates and records individual logistics activities data including order entry, inventory assignment, order selection, shipping, pricing, and invoicing and customer inquiry.

Main activities in a transaction processing system (TPS) can be divided into five categories including order management, order processing, preparations for distribution, transportation and shipping and, procurement. The most common tools and technologies that are used in the first layer include Electronic data interchange (EDI) and Electronic Fund Transfer (EFT), Extensible Markup Language (XML), barcode and Radio frequency identification (RFID). Application of some of these technologies such as RFID is increasing in pharmaceutical and healthcare systems for the purpose of tracking both medicines and patients in hospitals (12); other most commonly used technologies in this layer are contact wand, contact scanners, active and passive non-contacts canners, automatic identification and data capturing technologies, Freight Information and Tracking Systems (FITS), graphical information systems (GIS), Mobile satellite services, Radio determination satellite services, Global positioning systems, Cellular telephony systems, Short–range beacon systems (IR, Radio, MW), point of sale (POS) and Electronic Automatic Ordering Systems (EOS).

Management control system: the second level is management control systems that focus on measuring the performance, reporting, providing feedback and identifying exceptions. A management information system (MIS) helps to identify potential problems such as inventory short age which is critical for managing a supply chain.

Decision analysis systems: The third level is information systems for analyzing decisions. It includes programs that assist managers in identification, evaluation and comparison of different strategies and tactics. They may include modeling and analyzing tools, which can present a wide range of possible choices for the managers. The emphasis of Decision Analysis Systems shifted from efficiency toward effectiveness and they include decision support systems (DSS), enterprise resource planning (ERP), artificial intelligence application, and simulation/modeling systems (5).

Strategic planning systems: the last level focuses on information support systems to develop and refine the strategies used in a supply chain. It is typically more abstract, less structured and long-term focused. The executive information system (EIS) is the most common system in this layer. These systems display data graphically, and the information can be viewed from different angles in the mind have drill-downs and allow access to detailed information which can help to find relations and causes. EIS has brought the information of SCIS to the executive managers and allows executives to see the critical information at a glance (1) (13).

Several software, information systems and databases are being used in the health system of Iran to help better management of pharmaceutical and medical processes. The Pharmacies management software, the prescription registration software, the import database, the marketing authorization database, the price and sale database, physician's databases, hospital databases, the vaccines database, the pharmacies database and *etc* are some examples of the variety of software and databases which are being used in the Iran’s health system.

Several non-integrated and unprofessional information systems and databases are the reason for having a non-comprehensive and unreliable information flow in this area. As the past study result show that the information technology situations is not acceptable in Iran pharmaceutical industry(14). To improve this situation, the food and drug organization (FDO) aimed to develop a new information system to integrate all the information flowing in the pharmaceutical supply chain of Iran. "Iranian comprehensive information system for pharmaceuticals" which its acronym in Persian language is “SAJAD” was first proposed in 2007 and FDO hoped to put it into practice before 2016. However, it have not been implemented and in 2014 the new system which is called "track and trace" was introduced to information management in Iran pharmaceutical supply chain in general and control counterfeit medicines in Iran in particular. The substantial activities that have been considered in the first step for both above mentioned systems are online transactions, E-pedigree, the ability to track medicines, and E-Commerce. In the next step this system will get connected to e-prescribing and e-health dossiers. Main expected achievements of this information system include: better management of drug shortage, better planning for production, importing and stocking and also reducing fake drugs and counterfeits in the market (15).

## Discussion

SCISs (supply chain information systems) are used to coordinate information between internal and external customers, suppliers, distributors, and other partners in a supply chain. The most important sign for the success of any installed information system in a supply chain is how well this system has been able to support the activities of that supply chain, reduce buffer inventory stocks, reduce lead times, increase sales and improve customer services (10).

In order to achieve the highest performance level, supply chain strategies should be aligned with information systems strategies. In this situation, information systems meet the supply chain requirements in the best possible manner (16). However, not information technology values can be created only by using technology but applying managerial skills for using information plays an important role in value creation through IT. Moreover, the size of an organization, its successes, uncertainties, support of the senior management and the pressure applied by other partners play an important role in the effect that information technologies have on supply chains (17).

During the implementation of information technologies in a supply chain, organizations will usually face cultural and technical barriers. Overcoming cultural obstacles requires adapting to new behaviors, trust and mutual cooperation. Trust in any supply chain is necessary in order to share critical information such as costs, prices and etc. Cooperation between all business partners in a supply chain should be based on commitment in cooperation and sustaining that. In addition, the most important technical obstacles are providing security for information in the unsafe environment of Internet networks, lack of knowledge and technological infrastructures in organizations and heterogeneity of languages and technologies that are used in different systems which all must be dealt with. Moreover, data insecurity due to an insecure Internet environment is an important concern (2).

So, using IT applications can provide various benefits such as improving customer services, improving human resources efficiency, improving the total efficiency by allocating more time to more vital business activities, improving information quality and supporting collaborated planning and consequently improving the agility of the supply network. However, despite all benefits, there are some difficulties such as changing the business processes and business relations to adapt to the IT environment. In addition, organizations face more risks in an electronic supply chain including delay, system breakdown, lack of information security and *etc* (18).

Regarding the above mentioned risks and challenges it's a question that whether the "SAJAD" pharmaceutical supply chain information system or newly introduced system which is called "track and trace" will overcome facing cultural and technological obstacles or not.

From the technical perspective, there are at least two issues: online processing of all the pharmaceutical transactions does not seem to be easily achievable because IT infrastructures and networks are not available all over the country; for example the retail pharmacies in villages. And the other challenging issue in the information system is the lack of supporting levels for decision makings and also focusing only on the transaction levels. Although the transactional level is the hearth of an information system, the brain of the system should not be neglected.

In addition, some other issues can arise from social and cultural perspectives. Lack of skilled people can be the first issue and the main issue may be convincing all companies to share their real information on a unique system as a single entity. Maybe it would take a long time to conceive stakeholders that this information sharing can provide them more profits in the future (19).

 Furthermore, FDO as supervisor and the regulatory body is the system owner and will have access to all the data. Therefore, it is challenging to convince all stakeholders to share their real transaction information with FDO because of them being concerned about possible escalation of enforced laws by sharing the data with regulatory organizations.

## Conclusion

Supply chain plays an important role in creating competitive advantages for organizations and the success of any organization is linked to well managing its supply chain which can be achieved by using a proper information management system. Although the principles of a pharmaceutical supply chain are similar to other products, there are very specific issues which make it different. Pharmaceutical supply chains are not only important for organizations but are also important from social and political perspectives.

Although using information systems in supply chains may produce a lot of benefits, implementing them in supply chains is not easy and requires overcoming different technical and cultural obstacles. Newly introduced "Track and trace" system is the system that FDO hope to develop it as an integrated information system in the pharmaceutical supply chain of Iran. Besides all advantages and benefits of this system there are some important issues which should be carefully considered before starting to use it, such as the scope of the system, infrastructures, skilled people and creating trust between stakeholders. In the end, the proper design of all the levels of an information system and paying enough attention to barriers of performance and probable risks and challenges are highly recommended in order to be successful in establishing a prosperous information system.
